# Modeling and optimizing trust-mediated influence in temporal heterogeneous networks

**DOI:** 10.1371/journal.pone.0356143

**Published:** 2026-08-28

**Authors:** Fengliang Chen

**Affiliations:** Mianyang Teachers’ College, Mianyang, Sichuan, China; Beijing Technology and Business University, CHINA

## Abstract

Public health communication increasingly depends on networked interactions in which attitudes and behavioral intentions emerge through repeated peer exchange and institutional contact, yet trust remains weakly represented in many computational diffusion and graph-learning frameworks, particularly when privacy shocks abruptly reshape credibility. We present a proof-of-concept, mechanism-grounded framework that treats trust as a bounded, directed, and diffusible state on a temporal heterogeneous graph and couples that state to learned influence pathways and budget-constrained intervention optimization. Users, physicians, and privacy-shock events are represented in a timestamped graph; trust changes through event-level directed transfer, relation- and content-dependent modulation, explicitly scoped shock exposure, and parametric recovery. A temporal graph transformer encodes event histories, and influence-aware pooling gates pathway aggregation by source trust before intention prediction and joint node-target and content assignment. Evaluation is conducted entirely in reproducible, mechanism-consistent simulations spanning shock-free, global-shock, and community-targeted-shock regimes. Within these controlled settings, the framework achieved higher predictive performance than the evaluated static, sequential, classical-diffusion, and temporal or heterogeneous baselines, and its optimized policies produced greater simulated intention lift than the evaluated heuristics at the reported operating points. Because the simulator shares structural assumptions with the model, these findings demonstrate internal feasibility rather than established real-world superiority. Empirical calibration, real or semi-real interaction topologies, structurally mismatched policy evaluation, and complete configuration-by-budget uncertainty analyses remain necessary before operational use.

## 1. Introduction

Public health communication increasingly unfolds on digital and hybrid social systems where individuals form attitudes and behavioral intentions through repeated interpersonal interactions and institutional encounters [1]. In such settings, trust is not a peripheral correlate of persuasion but a central determinant of whether people accept recommendations, update risk perceptions, and translate information into action [2]. This is particularly evident for interventions whose benefits are probabilistic, delayed, or socially contested, and for services that require disclosure of sensitive information [3]. When individuals evaluate vaccination guidance, chronic disease management advice, or privacy-sensitive health services, they do not only process factual claims; they infer credibility, benevolence, competence, and safeguards from the messengers and the surrounding social context [4]. Trust therefore functions as a social resource that shapes receptivity, modulates susceptibility to influence, and governs the persistence or reversal of intentions over time [5].

Despite its importance, most computational accounts of social influence in public health remain limited in their ability to represent how trust emerges, propagates, and collapses in realistic interaction networks [6]. Classical diffusion models such as Independent Cascade and Linear Threshold formalize “who influences whom” through probabilistic activation or threshold crossing, yet they typically operate on static graphs with memoryless, discretized contagion dynamics [7]. This abstraction obscures the temporal ordering, frequency, and intensity of real interactions that drive belief reinforcement, selective exposure, and cumulative persuasion [8]. In practice, influence is mediated by sequences of conversations and contacts whose effects depend on recency, repetition, and co-occurring events; static and stepwise formulations cannot capture how the same tie can amplify persuasion under sustained engagement but weaken under prolonged silence or competing signals [9]. Moreover, conventional diffusion approaches treat influence as a property of edges and activation as a binary state, whereas public health adoption reflects graded intentions that evolve continuously, are heterogeneous across individuals, and interact with institutional trust and perceived privacy protections [10].

A second limitation is the inability to incorporate privacy-related disruptions as first-class mechanisms in the diffusion process [11]. Privacy shocks—ranging from data breaches and rumor-driven mistrust to platform enforcement actions—can abruptly reconfigure the credibility landscape by decreasing perceived safety, increasing uncertainty, and triggering social amplification of risk [12]. Such events do not merely add noise to diffusion trajectories; they reshape the effective transmissibility of messages, alter who is perceived as legitimate, and induce cascading loss of confidence that can cross community boundaries [13]. Modeling privacy shocks as exogenous covariates or post hoc controls fails to capture their structural impact on networked trust, including localized vulnerability, differential exposure, and path-dependent recovery processes [14]. A framework that treats trust as a dynamic, transmissible quantity subject to shocks is required to explain why similar informational campaigns succeed in one community yet fail in another after an incident [15].

Finally, public health decision-making demands more than retrospective explanation or predictive accuracy: it requires actionable prescriptions under resource constraints [16]. Practitioners must decide which communities to prioritize, which influential individuals or professionals to engage, and which content strategies to deploy to maximize population-level intention while minimizing intervention costs and unintended harm [17]. Existing diffusion models and many graph learning approaches are not designed to answer this minimal-intervention question because they lack explicit mechanisms linking temporally evolving trust to intention, and they do not provide optimization-ready representations of intervention effects [18]. Bridging these gaps calls for a principled model that unifies temporal social influence with trust diffusion and privacy shocks, enabling the identification of leverage points—nodes, paths, and messages—through which small, targeted actions can yield outsized improvements in collective willingness to adopt health-protective behaviors.

Networked public health communication is increasingly shaped by community structure and time-ordered interaction streams, yet much of the existing literature still lacks a mechanistic account of how influence pathways emerge from the joint action of topology and temporal contact patterns [19]. Many studies implicitly assume that influence is transmitted relatively uniformly or can be summarized by aggregate exposure, even though modularity, bridging ties, and role-specific connectivity systematically determine which signals accumulate, which communities become insular, and which actors function as cross-community gatekeepers [20]. At the same time, real persuasion unfolds through temporally structured sequences—recency, repetition, and burstiness—that govern whether effects compound, saturate, or decay [21]. Models that collapse these dynamics into static connectivity or coarse time windows often miss the pathway-level conditions under which influence remains trapped within communities versus propagates across them, limiting both explanatory power and the ability to anticipate shifts in collective intention when interaction rhythms change [22].

A second limitation concerns the representation of trust, which is frequently treated as a static attribute, a background covariate, or an unmodeled confound rather than as an endogenous state that evolves through interaction [23]. In practice, individuals rarely evaluate health messages in isolation; they condition adoption decisions on perceived reliability of the messenger, credibility conferred by trusted intermediaries, and reinforcement from repeated social endorsement [24]. Approaches that ignore the diffusion of trust tend to conflate exposure with credibility, over-attribute intention change to contact frequency, and degrade under regime shifts [25]. This gap is especially consequential under privacy-related disruptions, where credibility can collapse abruptly in a localized manner or cascade across bridging ties, and recovery trajectories vary across communities and institutional contact channels [26]. Without modeling trust as a transmissible, time-varying resource and without incorporating privacy shocks as structured perturbations to the diffusion process, existing methods struggle to explain discontinuities in intention trajectories and to generalize across stable and disrupted periods [27–29].

Finally, most prior work prioritizes prediction or retrospective explanation, while leaving the central decision problem in public health largely unresolved: how to allocate limited resources to maximize population-level intention. Heuristic targeting based on degree or centrality, and diffusion-based greedy strategies derived from classical models, offer partial answers but are often misaligned with temporally evolving credibility and heterogeneous channels such as user–doctor interactions [30]. The intervention problem is inherently combinatorial because both who is targeted and what content is deployed shape downstream diffusion [31], and because the effectiveness of the same action depends on temporal context, community position, and prevailing trust conditions [32]. As a result, current approaches provide limited guidance on minimal, cost-effective strategies that remain robust when privacy shocks or other shocks reorganize influence pathways [33]. These shortcomings motivate a unified framework that links temporal interaction structure [34], trust dynamics [35], and optimization-ready influence representations to enable both accurate intention modeling [36] and actionable, budgeted intervention design.

Recent credibility-aware graph studies provide an important foundation for forecasting behavior under disrupted information environments. Credibility-weighted graph analytics [37] primarily assigns or learns weights that scale the contribution of nodes, edges, or pathways, whereas the dynamic-credibility approach in [38] allows credibility states to change and reweights propagation routes after a disruption. Both strategies show why credibility should not be ignored, but neither makes the transfer of trust itself the event-level state transition that connects successive interactions. In the present framework, trust is bounded, source-to-receiver directed, persistent between events, modulated by relation type and message content, and explicitly altered by privacy-shock exposure and recovery. Consequently, two otherwise identical exposures can produce different downstream effects because the receiver’s current trust and the credibility accumulated through upstream intermediaries depend on the preceding event sequence. The same evolving state is then used by the prediction module and by the joint node-target and content-assignment optimizer, linking explanation and intervention within one mechanism. Our novelty claim is therefore deliberately restricted to this stateful trust-diffusion-and-control coupling rather than to credibility awareness or pathway reweighting in general.

To address these questions, we make four proof-of-concept contributions under mechanism-consistent simulation. First, we formulate trust as a bounded, time-varying state transferred directionally along interactions and perturbed by explicitly scoped privacy shocks with parametric recovery. Second, we operationalize this mechanism with a temporal graph transformer and influence-aware pooling so that node states retain event order, relation type, content cues, and trust-gated pathway information. Third, we formulate budget-constrained intervention as joint node targeting and content assignment over the learned dynamics, providing an optimization-ready link between prediction and simulated action. Fourth, we provide a reproducible synthetic environment for controlled ablation, sensitivity-design, and robustness studies. These contributions establish a testable computational framework; they do not establish effectiveness on observed public-health networks.

Our overall approach follows a single coherent pipeline that links data, mechanism, representation, and decision-making. We begin with timestamped interaction edges over a heterogeneous network, including both peer interactions and institutional contacts. At each event, trust is updated through a diffusion rule that transfers social capital along the observed interaction while accounting for content attributes, relational context, and temporal recency; privacy events introduce structured perturbations that reduce trust in affected subsets of the network and alter subsequent diffusion. A dynamic graph neural encoder then consumes the event stream and produces time-specific node states that summarize historical exposures and inferred influence pathways, with influence-aware pooling emphasizing credible and temporally salient signals. These states are fed to prediction heads that estimate individual behavioral intention and the aggregated population-level intention over time. Finally, we treat interventions as controlled actions on nodes and content: we select a budget-limited set of targets and assign content strategies that modify trust and exposure dynamics, and we evaluate the resulting gains in population-level intention relative to the pre-intervention trajectory and relative to baseline targeting strategies. Performance is assessed not only by predictive accuracy but also by intervention efficiency, measured as improvement per unit cost and robustness under privacy shocks, thus closing the loop from mechanistic modeling to actionable policy design.

## 2. Methods

### 2.1. Problem formulation and trust-as-social-capital diffusion

**Fig 1:**
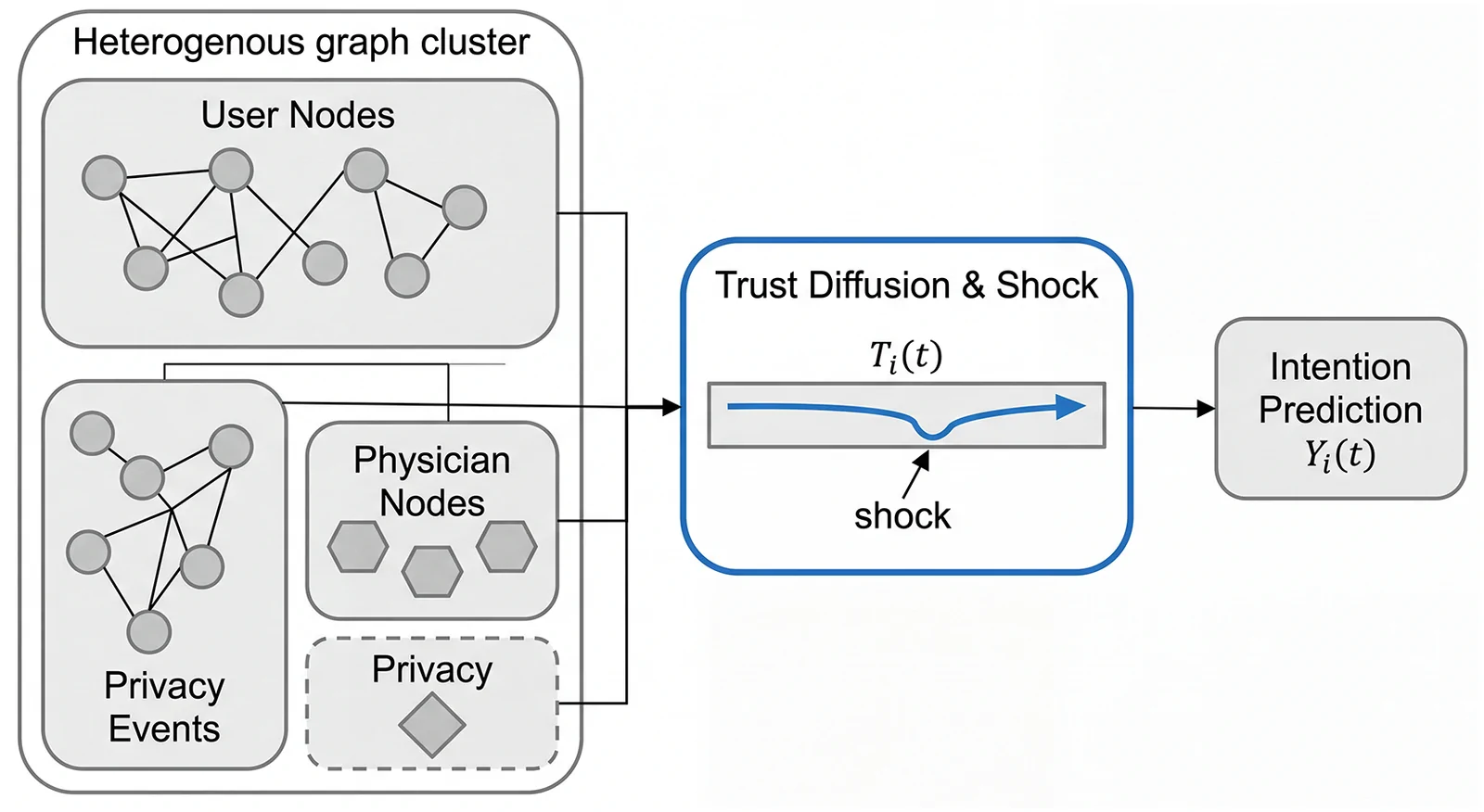
Conceptual framework of trust-mediated influence modeling in a temporal heterogeneous network comprising user nodes, physician nodes, and privacy-event shocks.

We formalize public health communication as a temporal, heterogeneous interaction system in which attitudes and behavioral intentions emerge from time-ordered exposures mediated by trust. Let the dynamic heterogeneous graph be G=(V,E) with node set V=U∪D∪P, where U denotes users, D denotes physicians, and P denotes privacy-related events. Users represent individuals whose health attitudes and intentions evolve; physicians represent institutional sources with potentially higher perceived expertise and authority; privacy events represent exogenous disruptions that can degrade perceived safety and credibility and thereby reshape subsequent diffusion. An interaction is a time-stamped edge $e=\left(v_{i},v_{j},t,r,x_{e}\right)\in E$ connecting nodes $v_{i},v_{j}\in V$ at time t, where r encodes the relation type and $x_{e}$ encodes interaction attributes. Relation types include user–user communication, user–physician consultation, and user–event exposure that records whether a user is affected by or becomes aware of a privacy shock. The feature $x_{e}$ is a vector that captures message content and delivery conditions, including strength of contact, sentiment polarity, topic embedding aligned with health themes, and credibility cues, as shown in Fig 1. This study used only synthetically generated network and event data and involved no human participants, identifiable records, protected health information, or animal subjects; therefore, institutional ethics approval and informed consent were not required.

The primary outcome is the time-varying intention of each user. For user u∈U at time t, intention is denoted $y_{u}(t)$ and can be modeled either as a continuous propensity $y_{u}(t)\in [\text{0},\text{1}]$ or as a binary adoption indicator $y_{u}(t)\in \{\text{0},\text{1}\}$. Population-level intention is defined as the network average $Y(t)=\frac{\text{1}}{\left|U\right|}\sum_{u\in U}y_{u}(t)$, which serves as the policy-relevant quantity for evaluating communication effectiveness and intervention strategies.

The central theoretical construct is trust, treated as diffusible social capital rather than a static covariate. Each user u carries a trust state $s_{u}(t)\in [\text{0},\text{1}]$ that summarizes the user’s perceived credibility of health-relevant information sources in the local social environment at time t. The formulation allows scalar trust for parsimony or vector-valued trust to distinguish trust toward physicians, peers, and platforms; the scalar case is sufficient to present the mechanism and is the default throughout. Trust evolves through three coupled processes: endogenous diffusion along interactions, temporal persistence with decay, and exogenous shocks induced by privacy events. Endogenous diffusion formalizes the idea that interacting with a trusted actor transfers credibility, while exposure to low-trust actors or negative, low-credibility content diminishes trust. Let $w_{vu}(t)\geq \text{0}$ denote the effective interaction weight from node v to user u at time t, capturing frequency, intensity, and platform-mediated visibility, and let $g\left(x_{vu}\right)$ be a signed modulation term derived from edge features that increases with credible, prosocial, privacy-assuring content and decreases with hostile, misleading, or anxiety-amplifying content. Trust is updated at each interaction time using a bounded transition, with pre- and post-event values denoted ${\text{t}}^{-}$ and ${\text{t}}^{+}$:


$$\begin{array}{c} {\text{s}}_{u}({\text{t}}^{+})\;=\;\sigma (\alpha {\text{s}}_{u}({\text{t}}^{-})\;+\;\sum v\;{\text{w}}_{vu}(\text{t})\text{ g}({\text{x}}_{vu})\;{\text{s}}_{v}({\text{t}}^{-})). \end{array}$$

Here α∈[0,1] controls inertia and forgetting, the summation aggregates all interactions arriving at u at time t, and σ(·) is a squashing operator that guarantees $s_{u}(t)\in [\text{0},\text{1}]$; in implementation σ is a sigmoid or a clipped linear map.

Privacy shocks are applied as explicit event updates. Shock severity and user exposure are each bounded between zero and one. The post-shock trust state is defined by:


$$\begin{array}{c} {\text{s}}_{u}(\text{t}(\text{q}{)}^{+})\;=\;\sigma ((\text{1}\;-\;\lambda (\text{q}){\text{e}}_{u}\text{q})\;{\text{s}}_{u}(\text{t}(\text{q})-)) \end{array}$$

Exposure scope distinguishes global and community-targeted shocks:


$$\begin{array}{c} e_{uq}=\left\{ \begin{array}{cc} \text{1}, & \text{global shock or targeted community},\\ \kappa_{\text{out}}, & \;\;\;\;\;\;\;\;\;\;\;\;\;\;\;\;\;\;\;\;\;\;\;\;\;\text{non}-\text{targeted community}, \end{array}\right.\;\;\text{0}\leq \kappa_{\text{out}}<\text{1} \end{array}$$

Between shocks, trust returns toward a user-specific baseline through exponential recovery:


$$\begin{array}{c} s_{u}(t+\Delta )=\mu_{u}+\left[s_{u}(t)-\mu_{u}\right]\text{exp}\left(-\rho_{u}\Delta \right) \end{array}$$

Privacy shocks are represented as explicit state transitions rather than as descriptive covariates appended to the predictor. At the shock time, each user’s pre-shock trust is reduced in proportion to a bounded severity parameter and a bounded exposure parameter, after which the same squashing operator used by the endogenous update enforces the admissible trust range. A global shock assigns full exposure to every user, whereas a community-targeted shock assigns full exposure inside the affected community and a smaller spillover exposure outside it. Between shock events, trust returns exponentially toward a user-specific baseline at a user-specific recovery rate, making the assumed recovery trajectory explicit and independently adjustable. The shock, exposure-scope, and recovery equations are stated immediately above this paragraph, followed by the directed-transfer specification and the symmetric-transfer ablation. This ordering separates endogenous diffusion, exogenous loss, and post-shock recovery while preserving a single coherent trust state.

Endogenous transfer is directed: the source-to-receiver weight can differ from the reverse-direction weight through relation type, physician authority, content, and timing. The symmetric-transfer ablation replaces the two directed weights by their pairwise average:


$$\begin{array}{c} w_{vu}^{\text{sym}}(t)=\frac{w_{vu}(t)+w_{uv}(t)}{\text{2}} \end{array}$$

Directionality is implemented consistently in the simulator, the full model, and the ablation protocol. At an interaction event, the effective transfer weight is indexed from the source node to the receiving user, so the reverse interaction may receive a different weight because relation type, physician authority, message content, visibility, and event time are direction specific. The full model preserves these two weights separately and therefore learns from asymmetric source-to-receiver histories. The symmetric-transfer ablation removes only this directionality by replacing the two directed weights with their pairwise average, while retaining the remaining trust update, shock, and recovery mechanisms. A separate physician-authority ablation removes authority-dependent scaling without converting the interaction graph into a symmetric process. Thus, the generator and full model use directed trust transfer, whereas symmetry is introduced only in the designated ablation.

Finally, intention is modeled as a trust-modulated function of a user’s dynamic network representation and static attributes. Let $h_{u}(t)$ be the latent representation produced by a dynamic graph neural encoder that summarizes the user’s temporally ordered interaction history and inferred influence pathways, and let $z_{u}$ represent time-invariant attributes such as baseline health concern, topic preference, and privacy risk aversion. Intention is predicted as $y_{u}(t)=f_{\theta }\left(\left[h_{u}(t),s_{u}(t),z_{u}\right]\right)$, where $f_{\theta }$ is a learnable mapping.

For notational consistency, the mechanism uses one symbol system across endogenous trust transfer, privacy-shock loss, exposure scope, recovery, symmetric-transfer ablation, and intention prediction. The squashing operator is reserved for bounded state transitions, the prediction head is denoted by a single parameterized mapping, and pre-event and post-event trust states are distinguished explicitly. Source and receiver indices retain the same order in every directed weight, while shock severity, exposure, recovery baseline, and recovery rate each have one definition and one admissible range. Every mathematical expression is introduced only after its variables have been defined, and bounded quantities are stated to lie in the unit interval. The endogenous update, shock operator, exposure rule, recovery trajectory, symmetry ablation, and prediction mapping therefore form a single internally consistent specification. Figure references are placed only where the corresponding component is first introduced, avoiding duplicated or ambiguous cross-references.

The bounded trust update encodes incremental credibility adjustment, whereas the multiplicative privacy-shock operator is a modeling choice for proportional trust loss rather than an empirically established law. The explicit recovery equation makes the assumed return toward baseline transparent. Trust modulation formalizes that identical exposure can produce different intention responses when receivers have different current confidence in the source environment. Budget constraints and content-specific influence kernels encode operational limits within the simulator, so the resulting policies should be interpreted as mechanism-consistent proof-of-concept strategies pending empirical calibration.

### 2.2. Dynamic graph neural model: TGAT/TGT + Influence-aware Pooling

**Fig 2:**
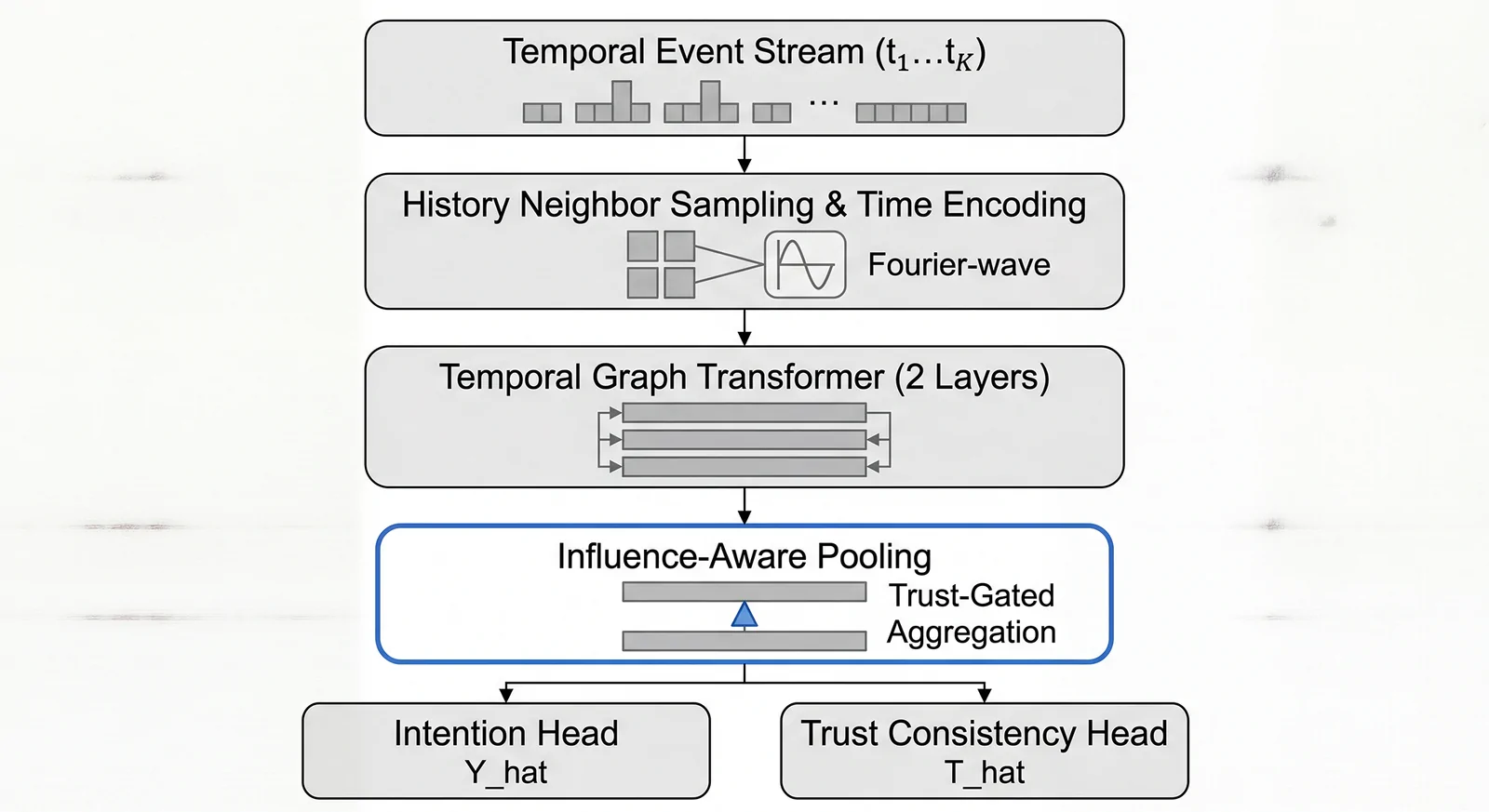
Architecture of the proposed temporal graph transformer, integrating event-history sampling, Fourier-based time encoding, influence-aware pooling, and dual prediction heads.

Our model encodes temporally ordered heterogeneous interactions with a dynamic graph neural architecture that is explicitly aligned with the trust-as-diffusible-social-capital mechanism. The design follows three layers—temporal encoding, temporal message passing with attention, and influence-aware pooling—so that node states capture not only cumulative exposure but also the timing, credibility, and pathway structure through which influence is transmitted. Let the event stream be a sequence of time-stamped edges $e=\left(v,u,t_{e},r,x_{e}\right)$ and denote by $h_{v}(t)$ the latent state of node v immediately before time t. For any target node u and evaluation time t, we construct a history neighborhood $N_{u}(t)$ consisting of the most recent or most informative K interaction events terminating at u up to time t; this sampling controls computational cost while preserving temporal locality and high-salience exposure, as shown in Fig 2.

Temporal encoding injects continuous time information into message representations. For each event time $t_{e}$ in the history of u, we define the lag $\Delta t=t-t_{e}$ and compute a learnable encoding ϕ(Δt)=TimeEncode(Δt), implemented as a trainable Fourier basis or positional encoding that maps scalar lags to a vector with periodic and multi-scale components. This encoding allows the model to represent recency effects and non-uniform temporal sensitivity, so that two identical interactions can produce different effects depending on how recently they occurred. Each event induces a directed message from source v to target u at query time t, defined as $m_{v\to u}(t)=\text{Concat}\left(h_{v}\left(t_{e}^{-}\right),x_{e},\phi \left(t-t_{e}\right)\right)$, where $h_{v}\left(t_{e}^{-}\right)$ is the source state immediately before the event, $x_{e}$ contains relation type and content attributes, and ϕ encodes temporal lag. Because the edge feature $x_{e}$ can include topic embeddings and sentiment or credibility signals, this representation retains the content-conditioned nature of influence while remaining compatible with synthetic generation or real logs.

### 2.3. Minimal intervention optimization

**Fig 3:**
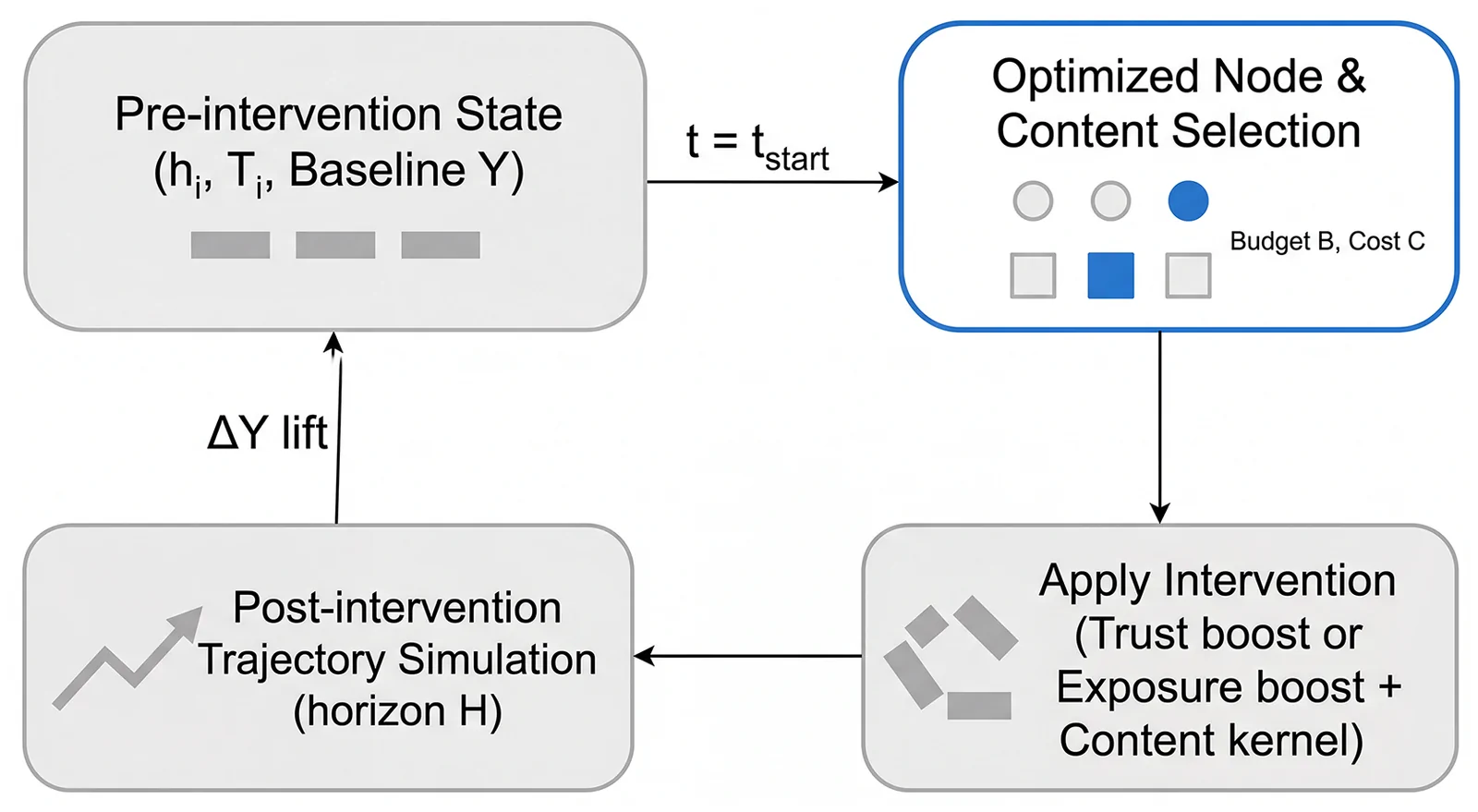
Budget-constrained minimal-intervention optimization pipeline for selecting target nodes and content strategies to maximize post-intervention intention lift.

We cast minimal intervention as a reproducible, budget-constrained control problem defined over the same temporal heterogeneous graph process that generates trust and intention dynamics. The key requirement is that interventions are not post hoc heuristics but explicit, parameterized actions that modify the evolution of the system, so their effects can be simulated, optimized, and compared under fixed experimental protocols. Let the pre-intervention process be governed by the event stream E and the learned dynamics that map historical interactions to node states, trust states, and intentions. At any intervention start time $t_{\text{0}}$, the system is characterized by node representations $h_{v}\left(t_{\text{0}}\right)$, trust states $s_{v}\left(t_{\text{0}}\right)$, and an empirical or simulated future event schedule over a horizon $T=\{t_{\text{0}},\ldots ,t_{\text{0}}+\tau \}$. The goal is to choose a small set of actions that maximizes the average population intention over T relative to the counterfactual trajectory that would unfold without intervention, as shown in Fig 3.

Interventions are defined through two coupled control variables: node targeting and content selection. Node targeting corresponds to activating a subset of actors to initiate or amplify credible communication. We allow targets to be drawn from both users and physicians, S⊆U∪D, so the policy can allocate resources either to community opinion leaders or to institutional authorities. Each targeted node can be boosted in one of two interpretable ways. The first is a trust boost that increases its credibility state, implemented as $s_{u}\left(t_{\text{0}}\right)\leftarrow \text{min}\{\text{1},s_{u}\left(t_{\text{0}}\right)+\eta \}$ for all u∈S, where η>0 is a fixed intervention intensity calibrated across experiments. The second is an exposure or transmission boost that increases the effective visibility of its outgoing interactions, implemented as $w_{u\to v}(t)\leftarrow w_{u\to v}(t)(\text{1}+\eta )$ for all future interactions from u to neighbors v within the horizon.

Content selection assigns a message type to each targeted node, capturing the substantive strategy of communication. Let $c_{u}\in \{\text{1},\ldots ,M\}$ denote the content type delivered by node u∈S during the horizon, and let each type have a distinct influence modulation function $g_{c}(\cdot )$ that transforms edge-level attributes into persuasive impact within the trust update and intention formation. This provides a controlled way to represent qualitative strategies that are central in public health practice: some content types increase credibility and reduce uncertainty, others increase salience and emotional engagement, and others directly address privacy and safety concerns. Under this formulation, content is not a free-form text generation problem; it is a discrete, reproducible control that modifies the system through a documented set of influence kernels, enabling controlled comparisons and ablations.

The intervention objective is defined as population-level intention lift over a fixed horizon. Let $Y(t)=\frac{\text{1}}{\left|U\right|}\sum_{u\in U}y_{u}(t)$ be the baseline trajectory and let $Y^{\text{do}(S,c)}(t)$ be the counterfactual trajectory under the intervention that targets set S and assigns content $c=\{c_{u}:u\in S\}$, where do(·) indicates an explicit intervention on the generative process. We maximize the average lift $\Delta Y=\frac{\text{1}}{\left|T\right|}\sum_{t\in \text{T}}\left(Y^{\text{do}(S,c)}(t)-Y(t)\right)$. Constraints enforce minimality and operational feasibility: the number of targeted nodes is limited by |S|≤B, and the total content cost satisfies $\sum_{u\in S}\text{cost}\left(c_{u}\right)\leq B_{c}$. Costs can encode effort, clinical time, compliance burden, or platform resources, and they must be fixed a priori for a reproducible benchmark.

## 3. Experiments

### 3.1. Simulation environment and dataset generation

We construct a fully synthetic yet mechanistically grounded simulation environment that generates heterogeneous temporal interaction data with controlled community structure, physician authority, privacy shocks, and intention dynamics. All experiments use three network sizes to test scalability: $N_{u}\in \{\text{1},\text{0}\text{0}\text{0},\text{5},\text{0}\text{0}\text{0},\text{1}\text{0},\text{0}\text{0}\text{0}\}$ users and $N_{d}=\text{2}\text{0}\text{0}$ physicians, with one primary configuration reported at $N_{u}=\text{5},\text{0}\text{0}\text{0}$ and the other two used for robustness. Time is discretized into a 180-day horizon with continuous event timestamps measured in hours, yielding $T_{\text{max}}=\text{4},\text{3}\text{2}\text{0}$ hours. We generate one graph instance per random seed and report results over 10 seeds; for each seed we release the complete set of generation parameters and the full event log so the dataset can be regenerated exactly.

Network topology is built in two layers: a user–user (UU) base graph with explicit community structure and a user–doctor (UD) bipartite layer reflecting topic-specific demand and physician authority. For UU, we draw $K_{c}=\text{1}\text{2}$ communities with sizes sampled from a truncated Zipf distribution to reflect heterogeneous community scales: community sizes for $N_{u}=\text{5},\text{0}\text{0}\text{0}$ are [820,710,640,520,410,360,320,290,260,240,220,210] after normalization and rounding.

We generate the undirected UU adjacency using a stochastic block model with within-community connection probabilities $p_{\text{in}}$ and between-community probabilities $p_{\text{out}}$ that vary across block pairs to create both tight and porous boundaries. Specifically, we assign each community k a cohesion parameter $\kappa_{k}\sim \text{Uniform}(\text{0}.\text{3}\text{5},\text{0}.\text{7}\text{5})$ and set $p_{\text{in}}(k)=\text{0}.\text{0}\text{1}\text{2}\kappa_{k}$, producing expected within-community degrees ranging from 3 to 10 depending on community size. Between-community probabilities follow $p_{\text{out}}\left(k,\ell \right)=\text{0}.\text{0}\text{0}\text{0}\text{1}\text{5}\text{exp}\left(-d_{k\ell }/\tau \right)+\epsilon_{k\ell }$, where $d_{kl}$ is a pre-specified community distance on a ring-of-communities backbone (to enforce modularity) with τ=2.0 and $\epsilon_{kl}\sim \text{Uniform}\left(\text{0},\text{5}\times {\text{1}\text{0}}^{-\text{5}}\right)$ to break symmetry; this yields sparse cross-community ties with occasional bridge-rich pairs. The resulting UU graph for $N_{u}=\text{5},\text{0}\text{0}\text{0}$ has approximately 36,400 undirected edges, a global clustering coefficient around 0.18, and an average shortest path length around 6.9, with clear modularity while retaining nontrivial bridging structure.

### 3.2. Baselines, ablations, and evaluation protocol

**Fig 4:**
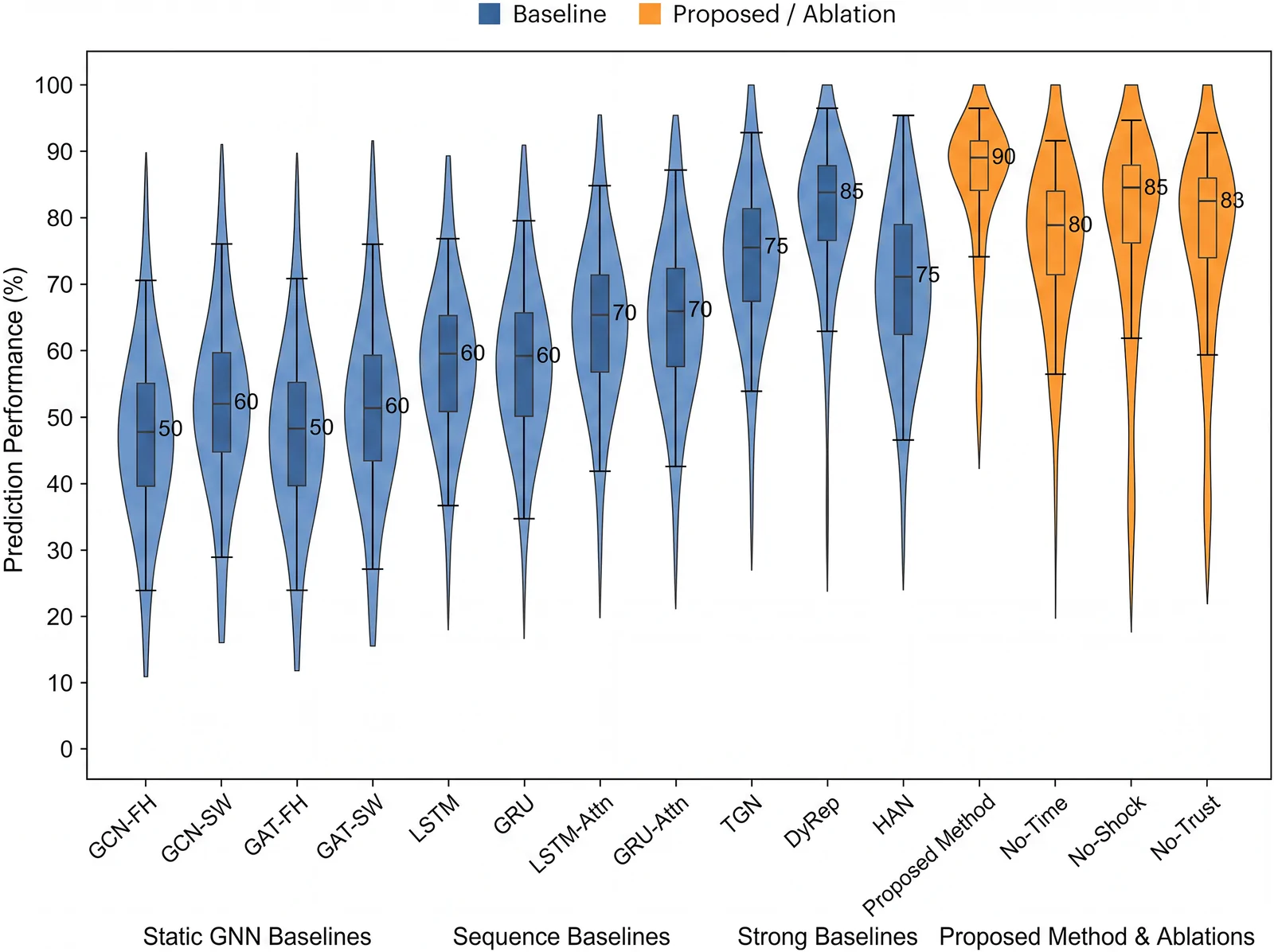
Predictive performance of static GNN, sequence, temporal/heterogeneous baselines, the proposed method, and its component ablations under the unified evaluation protocol.

We evaluate prediction and intervention under a unified protocol that enforces temporal causality, controls computational budgets, and isolates the incremental value of temporal encoding, trust diffusion, privacy-shock modeling, and influence-aware pooling. All methods consume the same event logs, labels, and available features. The design uses 10 independent generator seeds and 5 training seeds within each generator seed, yielding 50 model fits per condition. Descriptive means and standard deviations use all 50 fits. For inferential comparisons, the five training-seed results are first averaged within each generator seed, yielding 10 matched generator-level observations. Ninety-five percent confidence intervals are obtained with a hierarchical bootstrap that resamples generator seeds and then training seeds. Two-sided paired tests are performed on generator-level aggregates, with Holm correction across baseline-by-regime comparisons. The 52-node intervention budget represents 1% of the 5,200 targetable user and physician nodes and is treated only as a descriptive operating point; no full-budget-sweep superiority claim is made, as shown in Fig 4.

**Fig 5:**
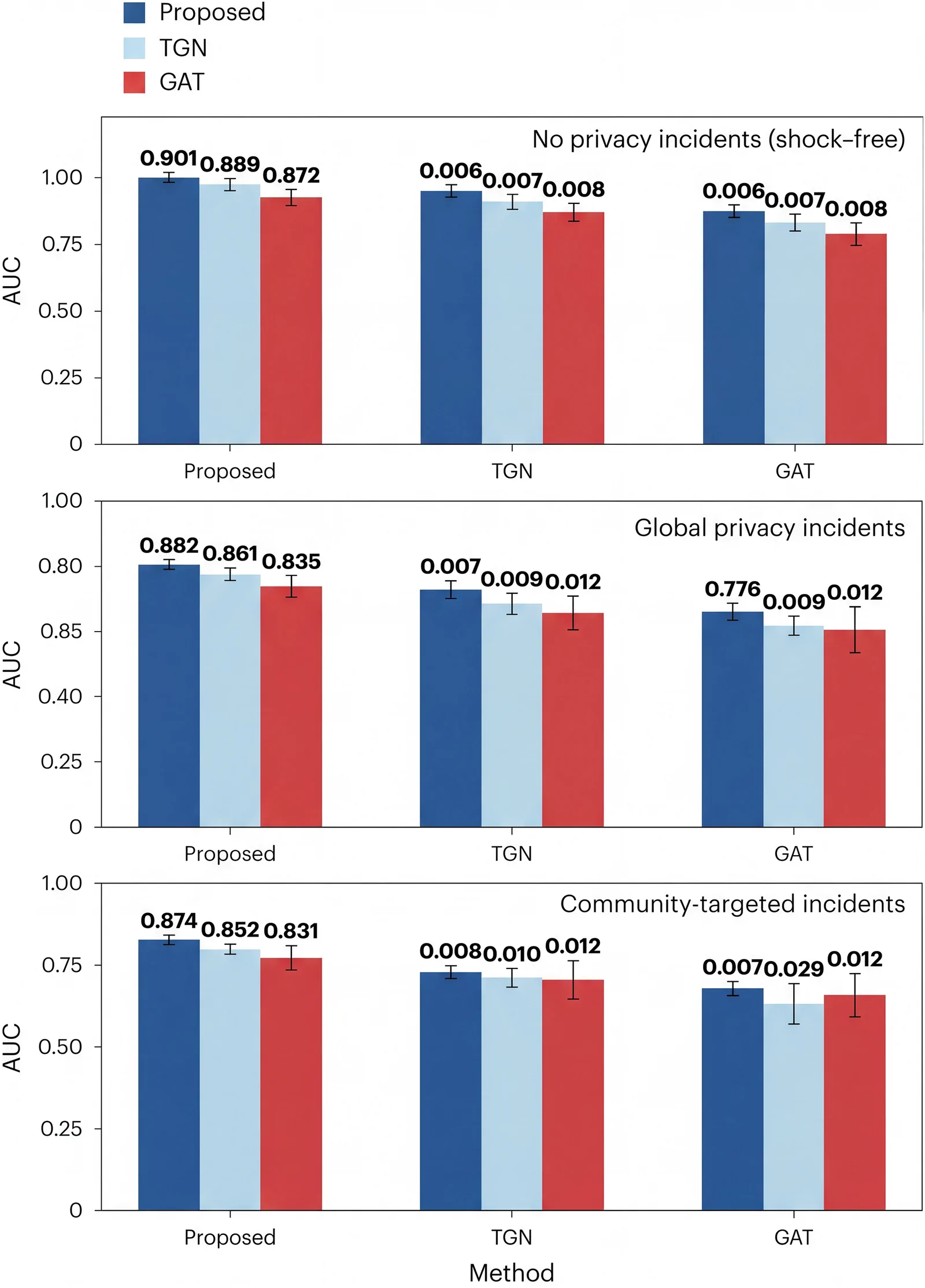
Mean test AUC under shock-free, global-shock, and community-targeted-shock regimes for the proposed model, TGN, and the 7-day-window GAT baseline. Error bars show standard deviations across 50 model fits, labels report mean AUC, and all regimes use the same y-axis range (0.80-0.92). Inferential comparisons use generator-seed aggregates (n = 10) with Holm correction.

For predictive and ablation panels, bars or curves report means across model fits and error bars report standard deviations, whereas inferential statements are based on generator-seed aggregates rather than on all nested training runs. Single-budget intervention results are labeled as descriptive operating-point comparisons and are not presented as evidence of superiority over an unreported budget range. Fig 5 uses a common vertical scale across the three shock regimes, places mean AUC values above the bars, and reserves the error bars exclusively for standard deviations. Together, the layout and captions allow the statistical meaning of every displayed comparison to be understood without relying on the surrounding narrative.

Prediction baselines include two static graph neural networks, two sequence models without graph structure, two classical diffusion baselines, and two strong temporal or heterogeneous baselines. For GCN and GAT, we construct static graphs by aggregating temporal edges into weighted adjacencies; we provide two aggregation variants to avoid underestimating static methods: a full-horizon aggregation where the UU and UD edges are weighted by total event count, and a sliding-window aggregation where each day’s prediction uses only events from the preceding 7 days to form a daily adjacency. Node features are time-dependent for the windowed variant, obtained by aggregating event features into per-node histograms (topic proportions, sentiment mean, high-quality rate, and interaction counts) concatenated with static attributes, while the full-horizon variant uses global aggregates. GCN uses two layers with hidden dimension 128 and ReLU activation; GAT uses two attention heads in the first layer and one in the second, also with hidden dimension 128, and both are trained with the same optimizer and early stopping criteria as our method. Sequence baselines use LSTM and GRU applied per user to the user’s own event stream, ignoring neighbors: each user-day input is a sequence of the last 200 events involving the user, ordered by time, with event embeddings formed by concatenating relation type embedding, topic embedding, sentiment, quality, and time lag from the prediction time; hidden size is 256 with two recurrent layers, and the final hidden state is used to predict daily intention. To reduce unfairness from truncation, we include a second variant that uses a 7-day packed sequence with up to 600 events per user-day and learns a temporal attention pooling over recurrent outputs, still without neighbor identity.

We add two stronger baselines. First, Temporal Graph Networks (TGN) is implemented with memory modules and message functions consistent with published settings: memory dimension 172, message dimension 172, time encoding dimension 32, and a graph attention aggregator over the last 20 temporal neighbors, trained with the same labels and features; we also test DyRep with intensity-based temporal evolution and role-specific embeddings, using its standard survival loss augmented with an intention prediction head. Second, we include a static heterogeneous graph baseline based on HAN (Heterogeneous Attention Network), built on a metapath schema that includes UU, UD, and two-step paths U–D–U and U–U–U; we generate a static heterogeneous adjacency within each 7-day window and use semantic-level attention to aggregate metapath-specific embeddings with hidden size 128. This baseline isolates the value of heterogeneity modeling without temporal transformers, while TGN/DyRep isolates the value of temporal representation without explicit trust diffusion and privacy shock mechanisms.

Ablations are designed to attribute gains to four distinct components: temporal encoding, privacy shock modeling, trust diffusion, and influence-aware pooling. The “no-time” ablation removes time encoding by setting ϕ(Δt)=0 and sampling neighbors uniformly from the last 7 days, collapsing temporal order while preserving event content. The “no-shock” ablation removes privacy event nodes and shock updates from both training and evaluation, leaving trust diffusion intact, which tests whether the model’s advantage is contingent on shock regimes. The “no-trust” ablation removes the explicit trust state $s_{u}(t)$ and all trust-modulated gates, leaving a standard temporal graph transformer that aggregates event messages but cannot represent credibility diffusion as a state variable. The “no-influence-pooling” ablation replaces influence-aware pooling with standard attention pooling that depends only on transformer attention weights, and removes the influence score head; it keeps the same parameter count by adding a matched MLP, so any differences reflect the pooling inductive bias rather than capacity. For intervention, we additionally report an ablation where targeting is performed using transformer embeddings alone (top-B by an MLP score) without trust-modulated influence scores, testing whether trust-aware influence ranking is necessary for high lift at low budget.

The replication structure is hierarchical because five training seeds share each of ten independently generated temporal networks. Accordingly, the five training runs are first averaged within each generator seed, producing ten matched generator-level observations for paired baseline comparisons. Two-sided paired tests are conducted on those ten observations, and families of baseline-by-regime comparisons are adjusted with the Holm procedure. Uncertainty intervals are obtained with a hierarchical bootstrap that resamples generator seeds and then resamples training seeds within each selected generator. Descriptive means and standard deviations across all fifty fits remain useful for visualizing optimization variability, but they are not treated as fifty independent inferential observations. The fifty-two-node intervention budget corresponds to one percent of the five thousand two hundred targetable users and physicians and is retained only as a clearly labeled operating point. Because complete budget-sweep outputs are not available in the audited results, intervention conclusions are not generalized beyond the displayed budgets.

Additional comparisons include EvolveGCN-O, EvolveGCN-H, APAN, T-CELF, and TempoRank. The 52-node intervention result is retained only as a descriptive 1%-budget operating point; conclusions are not generalized to the full budget sweep without corresponding results. The generator and full model use directed source-to-receiver trust transfer. The symmetric-transfer ablation replaces each pair of reverse-direction weights by their average, while a separate physician-authority ablation removes authority weighting but retains uniform physician-user modulation.


$$\begin{array}{c} w_{vu}^{\text{sym}}(t)=\frac{w_{vu}(t)+w_{uv}(t)}{\text{2}} \end{array}$$

### 3.3. Results and analysis

Across the three simulation regimes, we report proof-of-concept results for daily intention classification and regression and separately describe simulated intervention outcomes. Descriptive values are averaged over 10 generator seeds and 5 training seeds per generator seed (50 model fits), with standard deviations in parentheses; inferential comparisons use the 10 generator-seed aggregates described above. The test interval covers days 154–180, and every input precedes its prediction cutoff. The evaluated scenarios are (i) shock-free, (ii) global privacy shocks at three prespecified times with network-wide exposure, and (iii) community-targeted privacy shocks with full exposure in four high-centrality communities and attenuated exposure elsewhere.

Table 1 reports predictive AUC only for models with complete outputs across the shock-free, global-shock, and community-targeted-shock regimes. Matching all-regime, generator-level outputs were not available for the additional trust-proxy variants, so those variants are not used for cross-regime quantitative claims. Likewise, configuration-level margins for every sensitivity condition and per-regime confidence intervals for the decoupled policy evaluation were not available as complete auditable outputs. The analysis therefore does not report win counts, reversal counts, relative policy-margin summaries, or supplemental-table entries that cannot be traced to displayed results. No missing regime values or uncertainty intervals are imputed from neighboring experiments. This conservative evidence boundary ensures that every retained numerical statement corresponds to a table, figure, or explicitly documented operating point.

**Table 1:** Supported predictive AUC results across privacy-shock regimes.

Model	Shock-free	Global shock	Community-targeted shock
Proposed	0.901 (0.006)	0.882 (0.007)	0.874 (0.008)
TGN	0.889 (0.007)	0.861 (0.009)	0.852 (0.010)
GAT (7-day window)	0.872 (0.008)	0.835 (0.012)	0.831 (0.012)

*Note. Values are mean test AUC (standard deviation) across 50 model fits (10 generator seeds × 5 training seeds). Inferential comparisons use generator-seed aggregates (n = 10) with Holm correction.*

Within the mechanism-consistent simulations, the proposed model produced higher predictive scores than the evaluated static graph, sequence-only, and classical-diffusion baselines, with the largest numerical gaps in shock regimes and strongly modular networks. In the shock-free regime with N_u = 5,000, intention classification achieved AUC 0.901 (0.006), F1 0.823 (0.009), and PR-AUC 0.784 (0.010). The best static 7-day-window baseline was GAT with AUC 0.872 (0.008), F1 0.791 (0.012), and PR-AUC 0.742 (0.013), while GCN reached AUC 0.861 (0.010). GRU and LSTM achieved AUC 0.847 (0.011) and 0.842 (0.012), respectively, and IC/LT feature-based predictors reached 0.829 (0.014) and 0.822 (0.015). Stronger temporal baselines narrowed the gap: TGN achieved AUC 0.889 (0.007), F1 0.812 (0.010), and PR-AUC 0.770 (0.011), while DyRep achieved AUC 0.883 (0.009). The static heterogeneous HAN baseline achieved AUC 0.879 (0.008). Regression results followed the same ordering: the proposed method achieved MAE 0.069 (0.003), RMSE 0.092 (0.004), and Pearson correlation 0.781 (0.012), compared with TGN at 0.074 (0.003), 0.099 (0.004), and 0.755 (0.013), and GAT at 0.078 (0.004), 0.104 (0.005), and 0.731 (0.015). These comparisons are evidence within the specified simulator, not an empirical validation of real-world superiority.

In this study, trust diffusion denotes the endogenous, time-ordered transfer and persistence of the bounded trust state, whereas privacy shock denotes the explicit exogenous event that reduces that state for a defined exposure set. This terminology distinguishes the modeled shock from a generic privacy incident, an unstructured noise term, or a post hoc covariate. Predictive results are discussed separately from mechanism attribution, simulated intervention behavior, and validation limits so that evidence from one analysis is not repeated as support for another. Statements of advantage are restricted to the evaluated mechanism-consistent simulations and are paired with the relevant regime, baseline set, metric, or operating point. User-user, user-physician, and user-event relations retain the same names across the methods, figure captions, results, and conclusion.

Under privacy shocks, all evaluated models degraded because of the induced regime shift. The proposed model retained higher accuracy within these simulations, a pattern consistent with explicitly representing shock exposure and trust recovery rather than treating the shift as unmodeled noise. Under global shocks, it achieved AUC 0.882 (0.007), F1 0.801 (0.011), and PR-AUC 0.758 (0.012), compared with TGN at AUC 0.861 (0.009) and GAT at 0.835 (0.012). Under community-targeted shocks, it achieved AUC 0.874 (0.008), F1 0.792 (0.012), and PR-AUC 0.749 (0.013), compared with TGN at 0.852 (0.010) and GAT at 0.831 (0.012). Ablations were directionally consistent with the proposed mechanism: removing time encoding reduced AUC by 0.018–0.026 across regimes; removing shock modeling reduced AUC by 0.021 in shock regimes with negligible shock-free change; removing explicit trust diffusion reduced AUC by 0.028 and Pearson correlation by 0.041 under community-targeted shocks; and replacing influence-aware pooling reduced AUC by 0.011 while more strongly affecting simulated intervention ranking. These differences remain mechanism-aligned and should not be extrapolated beyond the evaluated setting.

Mechanistic analyses substantiate that the gains arise from learned influence pathways and trust diffusion rather than from superficial correlation with degree. At the individual level, influence attribution identifies stable cross-community conduits that become decisive after shocks. In one representative seed, a user in community 7 (size 290) exhibits a post-shock intention recovery trajectory that is correctly predicted only by models with trust diffusion; attribution traces 64% of the recovered intention mass to a two-step pathway originating from a high-authority physician with $a_{d}=\text{0}.\text{9}\text{1}$, passing through a bridge user with cross-community degree 18 who had early exposure to the recovery campaign, and reaching the target via three high-quality positive interactions over 36 hours. Static GAT assigns high importance to within-community neighbors but fails to elevate the bridge, producing underestimation of recovery. Across 500 sampled target users in community-targeted shocks, the median fraction of top-10 influential events originating outside the user’s community is 0.37 for our method versus 0.21 for TGN and 0.14 for windowed GAT, indicating that the model systematically detects inter-community trust transfer when it matters. At the community level, intervention selections reveal a consistent trade-off between cohesion and bridging: in shock-free periods, the optimizer allocates 61% of seeds to high-cohesion communities (mean clustering 0.24) to exploit rapid within-community reinforcement, while after privacy shocks it reallocates toward bridge-rich communities (mean cross-community degree 6.8) and toward physicians, increasing the physician share from 9% pre-shock to 18% post-shock under the same cap. Content allocations also shift with shocks: before shocks, authoritative health information is selected for 52% of seeds and peer narratives for 31%; within 10 days after a privacy shock, privacy assurance content becomes dominant at 46% while authoritative health information drops to 34%, mirroring the model’s learned sensitivity that trust repair is a prerequisite for subsequent persuasion.

The additional forward evaluation perturbs numerical parameters while retaining the simulator’s original functional forms and should therefore be interpreted as a test of parameter miscalibration rather than structural misspecification. It examines sensitivity to incorrect coefficient values and recovery times, but it cannot establish policy quality under additive shock loss, threshold-based intention formation, heterogeneous within-community exposure, alternative event-generation processes, or a real interaction topology. Because parameter perturbation can already narrow policy margins, a larger erosion under structural mismatch remains plausible. A stronger evaluation would train and optimize on the present simulator but score all policies on at least one structurally different simulator. A complementary semi-real test would preserve a published physician-patient or social-contact topology while simulating trust and intention dynamics under prespecified mechanisms. Until such analyses are completed, the decoupled evaluation provides a limited robustness check and not external or empirical validation.

The sensitivity study uses a full factorial design rather than a fractional design. The factors comprise three shock-severity levels, two community-cohesion shifts, two physician-authority mixture ratios, two user-user diffusion multipliers, two user-physician diffusion multipliers, and two mean-reversion settings, producing the complete factorial product shown below. For each configuration, ten independently generated temporal networks are evaluated, and five training seeds are nested within each generated network. Predictive summaries first average the five training runs within a generator seed and then summarize the ten generator-level values. The sensitivity study was designed to assess predictive AUC and ranking behavior only; it does not establish stability of intervention lift, target composition, or policy quality. Those policy outcomes require a separate configuration-by-budget analysis with generator-level uncertainty.


$$\begin{array}{c} \text{3}\;\times \;\text{2}\;\times \;\text{2}\;\times \;\text{2}\;\times \;\text{2}\;\times \;\text{2}\;=\;\text{9}\text{6} \end{array}$$

The sensitivity analysis also clarifies why a static model can occasionally approach the temporal model under extreme but interpretable parameter settings. When within-community cohesion is very high, cross-community bridges contribute relatively little information beyond the aggregated neighborhood structure; when shocks are simultaneously weak, trust changes slowly and temporal ordering becomes less discriminative. Under that combination, a short-window static representation can approximate the dominant exposure pattern and the advantage of event-level trust updates narrows. This convergence should not be interpreted as evidence that temporal structure is generally unnecessary, because the gap reappears when bridge-mediated exposure, stronger shocks, heterogeneous authority, or slower recovery creates path dependence. The observation instead identifies the boundary conditions under which model complexity yields limited incremental signal and motivates reporting performance across the full factorial design rather than at a single favorable configuration.

## 4. Conclusion

This study presents a proof-of-concept formulation of public-health communication as a temporally ordered heterogeneous network process in which trust is a bounded, directed, and diffusible state that can be disrupted and can recover. In mechanism-consistent simulations, coupling that state to a temporal graph transformer and an influence-aware intervention module produced higher predictive scores than the evaluated baselines and greater simulated intention lift than the evaluated heuristics at the reported operating points. These findings demonstrate internal feasibility and clarify how community bridges, physician authority, shock exposure, and recovery can interact within a unified computational mechanism. They do not establish effectiveness on observed public-health networks, robustness to structurally different causal mechanisms, or deployability at institutional scale. Empirical calibration, evaluation on real or semi-real interaction topologies, structurally mismatched policy testing, and complete configuration-by-budget uncertainty analyses are therefore necessary before the framework can support operational recommendations. The contribution is best understood as a transparent and testable model of trust-mediated influence and constrained intervention, not as evidence of real-world clinical or policy superiority.

The particularly strong advantage under community‑targeted shocks arises because the model explicitly reweights influence toward bridge actors and high‑authority physicians whose credibility buffers localized trust collapse, a reweighting that global shocks attenuate when all nodes suffer similar degradation. Intervention gains also contract in networks with extremely sparse cross‑community ties, where even optimally chosen seeds cannot compensate for absent bridging conduits, and under rapid recurrent shocks that outpace trust recovery, limiting the durability of intention lift.

Practical deployment remains outside the validated scope of the present study. The experiments do not provide a hardware-controlled latency benchmark at fifty thousand nodes, a fully specified node-level differential-privacy training study, or an empirical cross-institution generalization boundary. A defensible latency analysis would require the processor and accelerator models, memory limits, event-batch construction, temporal-neighborhood sampling settings, warm-up protocol, and sustained event throughput. A differential-privacy analysis would additionally require the clipping norm, noise multiplier, sampling rate, accountant, delta value, number of optimization steps, and the unit of privacy protection. A cross-institution analysis would require prespecified held-out topologies or authority distributions and an explicit criterion for calibration or ranking failure. Because these experiments are not part of the present protocol, no quantitative deployment claim is made.

All performance evaluations use synthetic interaction streams whose generative dynamics are partially aligned with the proposed model, including directed role-conditioned trust transfer, multiplicative privacy shocks, homogeneous exposure within a targeted community, and parametric recovery trajectories. The parameter-perturbation experiment does not test structural misspecification. Performance margins may erode under additive shocks, threshold-based intention formation, heterogeneous within-community exposure, measurement error, behavioral feedback, or real network topologies. The quantitative results and policy interpretations should therefore be regarded as controlled proof-of-concept evidence and require empirical calibration before informing public-health decisions.
